# Aceruloplasminemia: A Case Report and Review of a Rare and Misunderstood Disorder of Iron Accumulation

**DOI:** 10.7759/cureus.11648

**Published:** 2020-11-23

**Authors:** Chimaobi M Anugwom, Carlos G Moscoso, Nicholas Lim, Mohamed Hassan

**Affiliations:** 1 Gastroenterology, Hepatology and Nutrition, University of Minnesota, Minneapolis, USA; 2 Gastroenterology, University of Minnesota, Minneapolis, USA

**Keywords:** ceruloplasmin transferrin, liver damage, genetic metabolic disorders

## Abstract

Aceruloplasminemia is a rare disorder of iron accumulation inherited in an autosomal recessive fashion. It commonly presents as chronic microcytic anemia, and then progresses to signs and symptoms that are due to the accumulation of iron in multiple organs such as the brain, liver, pancreas, and thyroid. We present an asymptomatic patient with a history of microcytic anemia, who was evaluated for abnormal liver enzymes, and ultimately diagnosed with aceruloplasminemia.

## Introduction

Aceruloplasminemia is a rare autosomal recessive disorder of iron metabolism resulting in multi-systemic iron overload. It typically presents with mild chronic microcytic anemia, variable hepatic dysfunction, neuropsychiatric disturbances, and other sequelae of iron overload such as pancreas and thyroid disease. We present a patient with abnormal liver function tests and chronic microcytic anemia who was ultimately diagnosed with aceruloplasminemia.

## Case presentation

A 51-year-old female with a medical history of chronic microcytic anemia (baseline hemoglobin 10-11 g/dL) presented to the emergency department with new-onset post-prandial right upper quadrant pain. Laboratory evaluation showed an aspartate aminotransferase (AST) of 361 U/L, alanine aminotransferase (ALT) of 91 U/L, alkaline phosphatase (ALP) of 162 U/L, and total bilirubin of 0.6 mg/dL. Abdominal ultrasound showed cholelithiasis without cholecystitis or biliary duct dilation. Magnetic resonance cholangiopancreatography (MRCP) was obtained and was also negative for intra- or extrahepatic biliary dilation. The patient was admitted to the hospital and her liver enzymes continued to rise with AST 1422 U/L, ALT 1393 U/L, ALP 197 U/L, and total bilirubin of 0.3 mg/dL. Laparoscopic cholecystectomy was performed without complication and the patient was discharged home.

The patient was seen in the hepatology clinic with resolution of abdominal pain but persistently elevated liver enzymes. She was not jaundiced on physical examination, had no asterixis, and had no skin evidence of chronic liver disease such as spider angioma or palmar erythema. Additional laboratory evaluation revealed negative serologies for hepatitis A, B, and C, negative serologies for autoimmune hepatitis, and primary biliary cholangitis. Iron studies showed a low serum iron of 20 mcg/dL, a low iron saturation of 6%, and elevated serum ferritin of 7133 ng/ml. Subsequent testing for hereditary hemochromatosis was negative for mutations C282Y, H63D, and S65C. To ensure a full evaluation was completed for abnormal liver enzymes, serum copper and ceruloplasmin were also checked and were found to be undetectable. In addition, her 24-hour urine copper was noted to be <1 mcg/dL. Magnetic resonance imaging (MRI) of the liver revealed significant iron deposition in the liver. Percutaneous liver biopsy was performed, and histopathology showed marked hemosiderosis with accumulation of iron in hepatic parenchymal cells but no hepatic fibrosis.

**Figure 1:**
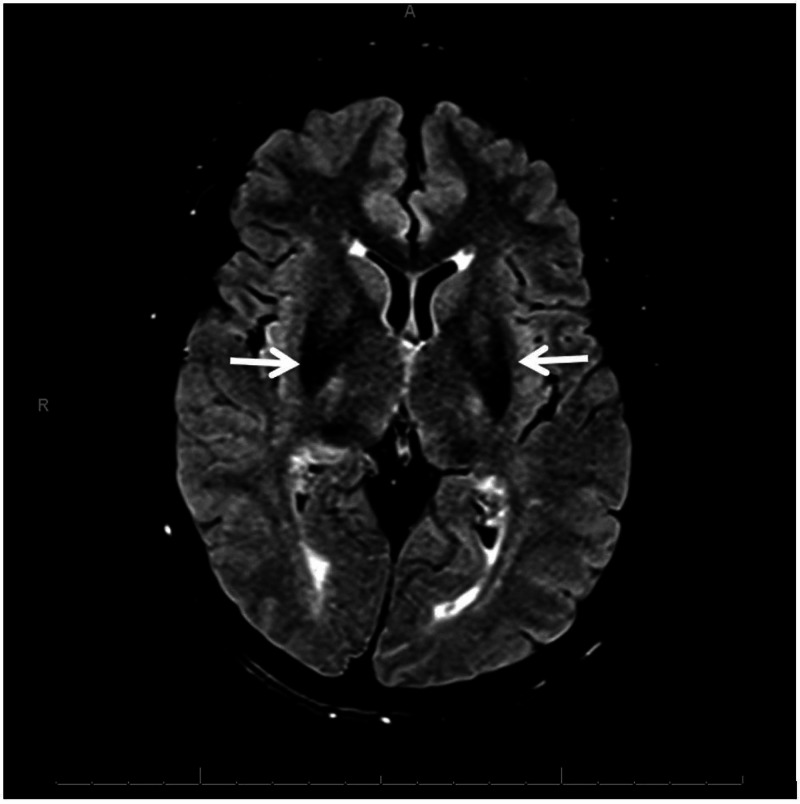
Brain magnetic resonance T2-flair images with prominent hypo-intense signal in the region of the basal ganglia (white arrows); A: anterior, R: right side

**Figure 2:**
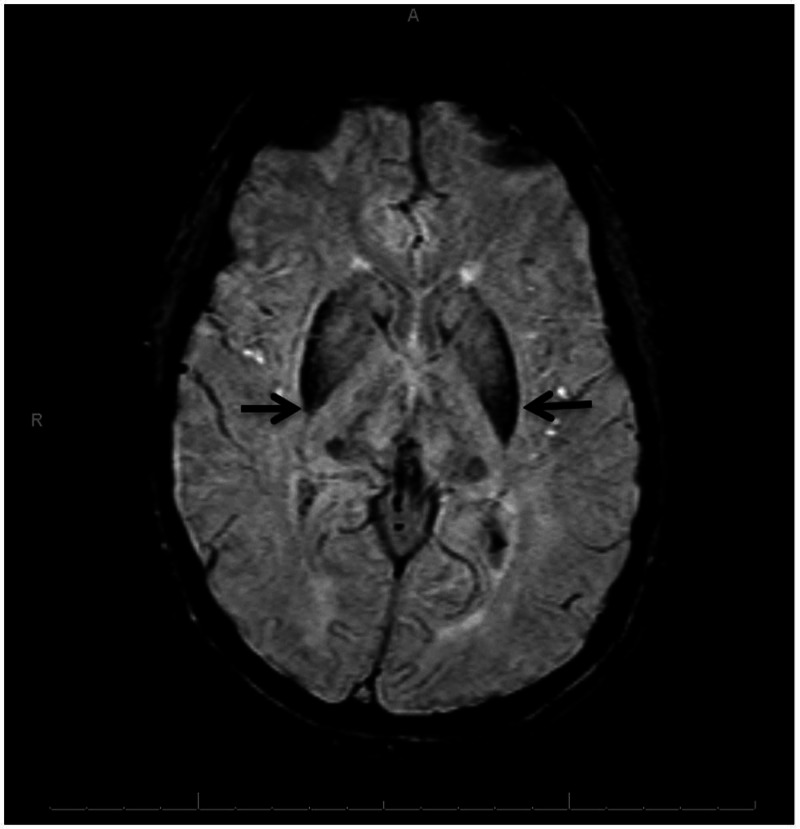
Brain magnetic resonance susceptibility weighted images (SWI) showing mildly increased iron deposition in the basal ganglia (black arrows); A: anterior, R: right side

The patient was then sent to ophthalmology for slit lamp examination which revealed the marginal appearance of Kayser-Fleischer rings. MRI of the brain was obtained to evaluate for iron deposition in the brain and was significant for mild excess mineral deposition in the basal ganglia, mid-brain, and dentate nuclei (Figures 1-2). Based on these findings, a diagnosis of aceruloplasminemia was entertained and the patient was referred to a genetic counsellor for discussion and testing. Using next-generation sequencing, two variants were detected in the CP gene - c.2149C>T (p.Gln717X), located on exon 12, and c.2972T>C (p.Ile991Thr), located on exon 17, both adjudged to be pathogenic. Next-generation sequencing was done with molecular confirmation of a diagnosis of autosomal recessive aceruloplasminemia.

The patient was referred for neurology and hematology consultations, as well as neuropsychological testing, all of which revealed no additional significant findings. Laboratory testing for thyroid dysfunction and diabetes mellitus have both been negative. Despite chronic mild microcytic anemia, the patient remains asymptomatic. Iron chelation therapy was discussed with her but she is being followed closely in the clinic without any therapy plans at this time. She was adequately counseled on the possibility of neurological symptoms in the future. There was no known diagnosis of aceruloplasminemia in her family and her family members have been referred for genetic counselling for discussion regarding further genetic testing.

## Discussion

Ceruloplasmin is a ferroxidase that can bind up to six atoms of copper. It plays a vital role in iron homeostasis, especially in the brain where it performs a neuroprotective function by safely oxidizing ferrous iron without the generation of free radicals, promoting iron efflux and reducing oxidative damage [1]. Although ceruloplasmin binds up to 95% of the body’s total copper in the plasma and is needed for copper to maintain its three-dimensional structure and enzymatic activity, it is not involved in copper metabolism [2].

Aceruloplasminemia is the hereditary absence of ceruloplasmin. It is a rare autosomal recessive disorder that occurs due to biallelic mutations in the CP gene located in chromosome locus 3q24-25 which result in systemic iron overload involving the eyes, pancreas, liver, and brain [3,4].

Ceruloplasmin, and other multicopper ferroxidases (MCFs) such as hephaestin, are vital to iron efflux from enterocytes, macrophages, hepatocytes, and neuronal cells [5]. Hephaestin is strongly expressed in the small intestine, whereas ceruloplasmin is located in other cells including those of the kidney, liver, and brain [6]. This function in the brain, where it plays a major role in regulating the uptake of iron by neuronal cells, thus reducing iron accumulation and oxidative stress, suggests that it confers a neuroprotective function [7]. In aceruloplasminemia, the inability to oxidize ferrous iron and promote binding to transferrin results in decreased iron delivery for erythropoiesis, which may be the putative mechanism for microcytic anemia in these patients [8]. Iron accumulation in the brain, beta cells of the pancreatic islets, and other visceral organs results in significant tissue damage, and this occurs in tandem with the loss of the antioxidant properties of ceruloplasmin, leading to unchecked production of deleterious oxygen species and resultant free-radical injury [9,10]. This mechanism has been demonstrated in studies where markers of lipid peroxidation from oxidative stress have been measured in patients with aceruloplasminemia [11].

Based on studies in Japan done by Miyajima et al., the estimated disease prevalence of aceruloplasminemia is estimated to be 1 in 2 million individuals born from non-consanguineous marriages [12], with most patients presenting between the ages 20 and 60. Outside Japan, however, the prevalence is largely unknown.

Miyajima described the classic triad of retinal degeneration, dementia, and diabetes based on observation of Japanese patients with aceruloplasminemia [13]. However, a much broader clinical presentation can be seen with cerebellar dysfunction, involuntary movements such as chorea, apathy, and memory loss being more common presenting symptoms [14]. Late-onset diabetes mellitus has been documented as a presenting syndrome, and this precedes the development of neurological symptoms [10]. Central hypothyroidism can occur from reduced production of thyrotropin-releasing hormone (TRH) related to iron deposition in the hypothalamus, but no other endocrine diseases have been reported to occur in patients with aceruloplasminemia [15].

Diminished or undetectable ceruloplasmin levels are also very suggestive of this disease although Wilson disease and Menkes disease must be considered in the differential diagnosis in the context of this finding [16]. The laboratory findings of microcytic anemia, low transferrin saturation, and high serum ferritin, as were apparent in our patient, are very common findings in aceruloplasminemia [14]. Her elevated liver enzymes were likely a result of biliary disease, making her diagnosis of aceruloplasminemia incidental. A retrospective review demonstrated that anemia is seen in 80% of patients and is often present in childhood, long before other neurologic symptoms develop [14,17]. Based on this clinical course, our patient remains at increased risk for neurological complications though it is difficult to predict the timing of development of these manifestations. 

Liver biopsy in these patients may be misleading as the presence of iron deposition in liver parenchymal cells may suggest HFE-hemochromatosis. However, cirrhosis from aceruloplasminemia is unlikely to occur and overt hepatic dysfunction and liver failure is rare [18]. Brain imaging may be elucidative and evaluation with MRI may demonstrate neurodegeneration in the thalamus, basal ganglia, and cerebellum [19]. Unequivocal diagnosis of aceruloplasminemia is achieved with genetic testing showing biallelic mutations in the CP gene. Since many cases of aceruloplasminemia are asymptomatic, diagnosis is dependent on a high index of suspicion especially with positive family history and mild laboratory abnormalities such as microcytic anemia.

There is limited data on the management of aceruloplasminemia and most available literature is based on case reports and case series. The mainstay of treatment is iron chelation, and though quite helpful in reducing iron in the liver parenchyma, it has a questionable impact on neurological manifestations [14]. Deferiprone is a frequently used chelating agent, which in contrast with deferoxamine and deferasirox, crosses the blood-brain barrier [16]. Iron chelation therapy may also be used in tandem with fresh frozen plasma to restore serum ceruloplasmin. The use of vitamin E or zinc has been studied with both medications having fleeting benefits [14]. Hayashida et al. published a case report demonstrating improved neurological status in a patient with aceruloplasminemia using minocycline [20]. Recombinant ceruloplasmin has been used in mice, revealing improved neurological function and the salvage of ferroxidase activity, however, this has not been replicated in humans with aceruloplasminemia [17].

## Conclusions

In conclusion, while our patient developed abnormal liver enzymes due to symptomatic biliary disease, the diagnosis of aceruloplasminemia was incidental. Although many cases of aceruloplasminemia are asymptomatic and depend on a high degree of suspicion to obtain a diagnosis, the presence of iron overload, in the setting of microcytic anemia and absent ceruloplasmin, should prompt genetic testing for this rare condition.
